# The Treatment of Syphilis by Subcutaneous Injection

**Published:** 1882-03

**Authors:** 


### Selections.

### The Treatment of Syphilis by Subcutaneous Injection.

The history of this mode of treatment is short but interesting, and its latest developments instructive and promising for good. Scarenzio, of Pavia, was the first to employ this method, about 1854, using calomel suspended in glycerine. (*Annales Universelles de Medicine, 1864.*) His formula was:

Calomel—0 gr. 20 ctgr. to 0 gr. 30 ctgr.

Glycerine (or Mucilage, or Water)—1 gramme.

M.—For one dose.

One or two such injections caused the lesions to disappear after eight to fifteen days. He published eight cases with good results, except that a few times abscesses were formed.

Scarenzio's method was taken up later on, in 1864–65, at the Venereal Hospital in Milan, by Dr. Ambrosoli, who fully corroborated Scarenzio's results. Later on, still in Italy, Riccordi, Monteforte, Soresina, and Bertarelli used the same, or similar, methods with satisfactory results.

In Great Britain, Berkely-Hill seems to have used, about 1866, injections of the corrosive chloride in eleven cases of syphilis, with a dose of one milligramme, and with good results. If the dose was increased, abscesses at point of puncture, salivation, colic and diarrhœa, were often found.

Lewin, at La Charite in Berlin, experimented in 1868 on a grand scale, using the corrosive chloride dissolved in water at a dosage of 5 to 10 milligrammes, adding morphia in some cases to make the injection less painful. His average number of injections was sixteen for each patient, and the average total quantity of the sublimate was 15 centigrammes for each. Results good.

M. Hardy tried the Berkeley-Hill and Lewin method on some patients at L'Hopital St. Louis in Paris, but gave it up on account of the severe pain, abscesses and eschars resulting from the injections. Diday, moreover, had the same results. Quite a number of physicians followed up the method during 1868–69; some liking it, others opposing it.

Liegeois communicated his results to the Paris Society of Surgery, at its meetings held on June 2d and 9th, 1869. His formula was as follows:

Distilled Water—90 grammes.
Sublimate—0.20 ctgrms.
Hydrochlorate of Morphia—0.10 ctgrms.
M.—To inject 1 gramme.

That is rather more than 2 milligrammes for each time; he found no inflammatory reaction at the wound. In two cases only a slight eschar followed. The pain was insignificant; four cases of slight salivation occurred in one hundred and ninety-six patients. Each patient had two injections every day. Average length of treatment, thirty-seven days, and relapse was noticed in 37½ per cent. He thought that the drug cured much more rapidly than if administered by way of the mouth. Liegeois advised the employment of this treatment in preference to all others, claiming that patients were perfectly competent to use the method themselves, without coming every day to the surgeon.

Aime-Martin presented his method to the Society of Medicine of Paris, on the 7th of August, 1868. He prefers a formula like the following:

> Double Iodide of Mercury and Potassium—0.40 centigrammes.
> Muriate of Morphia—0.05 centigrammes.
> Distilled Water—10 grammes.
> M.—Inject 10 drops every other day.

The morphia to be added at moment of injection, to avoid precipitation. Martin reports his cures as rapid and free from local lesions.

Bricheteau (*Bulletin de Therapeutique, 1869,*) uses this formula:

> Double Iodide of Mercury and Sodium—1 gr. 50 centigrammes.
> Distilled Water—100 grammes.
> M.—5 to 10 drops as an injection, once or twice a day.

1 gramme contains 1 centigramme of the mercuric salt.

Belhomme employs this formula:

> Double Iodide of Mercury and Morphia—0 gr. 50 centigr.
> Distilled Water—20 grammes.
> M.—5 to 10 drops once a day.

Bouchardat mentions (1872) the following solution (of Staub):

> Corrosive Chloride—1 gr. 25 centigr.

> Ammonium Chloride—1 gr. 25 centigr.

> Sodium Chloride—1 gr. 15 centigr.

> White of Egg—No. 1.

> Distilled Water—250 grammes.

> M.—

This is a great advance, and the mixture is called "Solution of the Chloro-Albuminate of Mercury" by its inventor.

Quite recently Mialke has shown that subcutaneous injections of the corrosive chloride of mercury cannot be absorbed, except when the metallic salt acts upon the albuminous contents of the connective-tissue spaces, and produces, at their expense, an "albuminate of mercury," which is easily absorbed. This coagulation in the connective tissue is the principal cause of pain, of formation of inflammatory nodules, or even of the sloughs we sometimes find. It is, therefore, reasonable to form the "albuminate of mercury" outside of the body, and avoid these annoyances (Terrillon, *Bulletin de Therapeutique*, 4th, 5th and 6th parts, 1880).

The thesis of Mr. Cotte (Paris, 1873,) may be referred to for interesting details on this point. This advance was not appreciated, and our journals are full of accounts of accidents following these injections. Nevertheless the art advanced, and surgeons sought after new compounds; thus, Cullingworth used the bicyanide in glycerine and water; Sigmund also advised a similar formula; Aime-Martin used the double iodide of mercury and potassium in the formula already given.

At last Bamberger used chemically pure peptone in combination with mercury. Martineau, in his excellent article, which I am using so freely (*Union Medicale*, No. 99, et seq., 1881,) does not say how to prepare his "peptone de viande" (it is erroneously printed "pepsine de viande," which is nonsense,) but Catillon (*Repertoire de Pharmacie* quoted by *Druggists' Circular*, January, 1881,) uses this formula:

Lean Beef, minced—2 pounds.
Hydrochloric Acid (sp. gr. 1.18)—5 drachms.
Water—10 pints.
Pepsine—sufficient.

Digest 12 hours at 45° C. with slight excess of pepsine. Keep temperature between 43° C. and 48° C.; agitate from time to time, and after two to six hours the mixtnre is nearly transparent. After 12 hours strain and filter. Saturate with bicarbonate of soda, and evaporate on water-bath till a pellicle forms on its surface. It then has, when cold, a sp. gr. of 1.15, and contains one-half of its weight of solid peptones.

Bamberger's formula was this:

Having made a solution of the sublimate in water of 5 per cent., and one of chloride of sodium of 20 per cent., he dissolved 1 gramme of "meat peptone" in 50 cubic centimetres of distilled water, and filtered it. He then adds to this last 20 cubic centimetres of the mercury solution, and after well mixing he adds just enough of the sodium chloride solution to dissolve the precipitate of peptonate of mercury, the amount required being about 15 to 16 cubic centimetres. Then add to the solution of "mercuric peptone" enough distilled water to make up 100 cubic centimetres, thus making it just one per cent. mercury—that is, 1 gramme of the solution represents 1 centigramme of the sublimate, about one-sixth of a grain. The solution is fit for use after settling some days, decantation and filtration. The dose is 1 cubic centimetre every three or four days; if oftener repeated, abscesses and salivation are to be feared.

Zeissl and Neumann, of Vienna, and Von Rinecker, of Würzburg, have used Bamberger's formula. Terrillon (op. cit. supra) used it also during his earlier experiments. Later on he used the following formula:

Biniodide of Mercury—1 gramme.
Potassium Iodide—1 gramme.
Tribasic Sodic Phosphate—2 grammes.
Distilled Water--Up to 50 cubic centim.

Both formulæ have produced some local lesions, painful nodosities, more or less persistent indurations, but no serious troubles like abscesses or sloughs. Other preparations, the lactate, phosphate, acetate, and biniodide of mercury, have been used, but no more successfully than those already noted.

Martineau, of the Hopital de Lourcine, has, since the 12th of April last, used the following formula:

Bichloride of Mercury—10 grammes.
Peptone de Catillon, dry.
Chloride of Ammonia, C. P. of each—15 grammes.

1 gramme contains 25 centigrammes of the sublimate.

This formula has given best results, because the Catillon peptone is exceptionally fine and pure; and since pure muriate of ammonia is the best solvent of albuminate of mercury which is so easily precipitated, it has also been found best to let the peptone be in excess slightly.

The following solutions have proved excellent:

Solution (A).

Ammoniated Mercuric Peptone—0 gr. 40 centigr.
Distilled Water—30 grammes.
M.—This contains 4 milligrammes of sublimate in 1 gr. 20 centigr., and will keep well for several days.

Solution (B) is more stable.

Mercuric Peptone (as above)—0 gr. 40 centig.
Distilled Water—25 grammes.
Neutral Glycerine—6 grammes.
M.—Dose and strength same as (A).

Solution (C) appears entirely stable.

Mercuric Peptone—0 gr. 40 centig.
Glycerine, C. P.—36 grammes.
M.—Same dose and strength as (A) and (B).

Since 12th of April, Dr. Martineau has treated 51 patients; the total number of injections up to June 21, 1881 (the date of his paper), is 751.

Series 1 consists of those who had 1 milligramme of mercury injected every three days.

In series 2 the dose was raised to 2 milligrammes every three days.

Series 3, 2 milligrammes every two days, and series 4 every day.

Series 5, 3 milligrammes every day.

Series 6, 4 milligrammes every day, and finally, series 7, five milligrammes every day—the last only since June 21, 1881, but is well supported, no pain, no local inflammation, and no mercurialization. He still proposes to go up to 6, 7, and 8 milligrammes per day, the result to be noted in another paper.

At the seance of the Academie de Medicine of Paris, on August 9, 1881, Dr. Martineau, in reply to questions, said that he had gone as far as 6 and 7 milligrammes of the sublimate every day, and had now used 1,300 injections, and yet never has had any salivation, although the mercury was freely eliminated by the kidneys, as shown by constant tests. He finds any local irritation very unusual, and prefers, as place of injection, the back, the sides of thorax, and some of his patients have had up to 45 injections, still without the least local accident. He finds the method superior to all others, especially in cases of severe syphilis, with serious symptoms in rapid evolution, and even up to 6 or 7 milligrammes per diem he has almost never pain, no abscesses or gastro-intestinal irritation, and rapid amelioration of symptoms. He justly remarks that it is yet too early to definitely pronounce upon the probability of relapses. Martineau's cases are highly interesting and valuable, but space will not permit their reproduction here. It is curious to note how rapidly the worst syphilitic accidents have disappeared under the hypodermic treatment, and in most cases without irritation or pain. It is very much to be desired that some American syphilographer should reproduce Martineau's experiments on a large scale in a hospital, as under such circumstances the method presents many incontestible advantages, even if it be not as well adapted to private practice.—*Annals of Anatomy and Surgery.*

